# Restoring well-being: quality of life improvements following dolutegravir-based therapy after antiretroviral failure

**DOI:** 10.1016/j.bjid.2026.105821

**Published:** 2026-05-16

**Authors:** Liliane Lins-Kusterer, Carlos Brites, Marcus Vinícius Guimarães de Lacerda, Kimberly Page, Fabianna Bahia, Estela Luz, Aline Ramalho, Sandra Wagner, Maria Belen Arriaga, CODE research group collaborators, Cristiani Stelitano, Valdez Madruga, Tânia Reuter, Alexandre Naime Barbosa, Rosa Dea Speahack, Monica Baumgardt Bay, Tâmara Newman, Karen Morejon, Maria Patelli, Fábio Leal, Cristina Murray-Krezan, Mary Carmody, Jess Anderson, Alejandro Aragon, Benjamin Chase, Fátima Dutra, Roberto Zajdenverg, Bryn Jones, Eduardo Sprinz

**Affiliations:** a Universidade Federal da Bahia/Fundação Bahiana de Infectologia, Salvador, BA, Brazil; b Fundação de Medicina Tropical Tropical do Amazonas, Manaus, AM, Brazil; c GSK Brazil, Rio de Janeiro, RJ, Brazil; d Centro Estadual Especializado em Diagnóstico Assistência e Pesquisa, Salvador, BA, Brazil; e Hospital Geral de Nova Iguaçu, Rio de Janeiro, RJ, Brazil; f Instituto Nacional de Infectologia Evandro Chagas, Rio de Janeiro, RJ, Brazil

**Keywords:** HIV, Combination antiretroviral therapies, Dolutegravir, Quality of life, Treatment failure, Patient-reported outcome measures

## Abstract

**Background:**

Dolutegravir-based antiretroviral therapy has improved virologic outcomes in people living with HIV; however, its effects on Health-Related Quality of Life (HRQoL), particularly among individuals with prior antiretroviral failure, remain insufficiently characterized. We aimed to compare HRQoL in people living with HIV initiating dolutegravir-based regimens, with or without previous antiretroviral failure.

**Methods:**

This multicenter study was conducted across 15 Brazilian HIV centers. Participants completed the RAND-36 questionnaire at baseline and after 12-months of dolutegravir-based therapy. HRQoL changes were assessed within groups and compared between groups using change scores over time.

**Results:**

A total of 439 participants were included in the longitudinal analysis, of whom 373 had no prior therapeutic failure and 66 had previous antiretroviral failure. Among participants with prior failure, significant improvements were observed in role-emotional functioning (Δ = 17.17; p = 0.010), social functioning (Δ = 10.79; p = 0.008), and pain (Δ = 10.66; p = 0.024), whereas HRQoL remained stable among individuals switching therapy without prior failure. Between-group comparisons demonstrated significantly greater improvements in role-emotional functioning, social functioning, pain, and general health among individuals with prior therapeutic failure.

**Conclusions:**

Switching to dolutegravir-based therapy was associated with stable HRQoL among individuals on suppressive regimens and with significant improvements among those with prior antiretroviral failure. These findings support the relevance of psychosocial and patient-reported outcomes in the evaluation of treatment response in people living with HIV.

## Introduction

While virologic suppression remains a central goal of Antiretroviral Therapy (ART), the assessment of patient-reported outcomes, particularly Health-Related Quality of Life (HRQoL), can improve the management of conditions associated with long-term treatment and comorbidities.^1^ Changes in HRQoL may capture not only clinical effectiveness but also broader psychosocial and functional well-being among individuals receiving long-term ART. Prior experiences of therapeutic failure may negatively influence HRQoL due to complex treatment histories, adverse drug effects, and cumulative psychological burden.

The RAND-36 questionnaire is widely used to assess HRQoL across multiple dimensions of functioning and well-being and provides a structured framework for evaluating the subjective impact of antiretroviral therapy on patients’ daily lives, encompassing physical, emotional, and social domains that may be influenced by treatment success or failure.^2^

Dolutegravir (DTG), a second-generation integrase strand transfer inhibitor, has potent antiviral activity, a high genetic barrier to resistance, and favorable pharmacokinetic characteristics.^3^ Previous studies have shown that switching to DTG-based regimens is associated with benefits in patient-reported outcomes, including physical and emotional well-being.^4^ However, these studies primarily evaluated populations on stable ART, without prior antiretroviral failure. Individuals experiencing ART failure may face challenges such as cumulative drug toxicities, diminished treatment optimism, and psychosocial distress, which can substantially affect HRQoL.

A prospective cohort study conducted in Spain reported significant improvements in quality of life, treatment satisfaction, and emotional well-being after switching to DTG-based regimens.^5^ Nevertheless, that study did not focus on individuals with documented therapeutic failure. Therefore, this study aimed to assess and compare changes in HRQoL over a 12-month period following the initiation of dolutegravir-based therapy in individuals without previous ART failure and in those who transitioned to DTG after documented virologic failure. Understanding these differences may provide clinically relevant insights into the broader impact of treatment strategies beyond virologic suppression, particularly among individuals with complex treatment histories, and may help inform patient-centered approaches to HIV care.

## Materials and methods

This prospective observational study was conducted between January 2021 and December 2024 across 15 AIDS clinics in 7 Brazilian states. Participating sites were distributed across multiple regions and included both specialized referral centers and outpatient HIV clinics, reflecting a heterogeneous population of people living with HIV. The study aimed to evaluate changes in Health-Related Quality of Life (HRQoL) among People Living with HIV (PLHIV) enrolled in an observational cohort that assessed clinical outcomes of PLHIV initiating Dolutegravir (DTG)-based Antiretroviral Therapy (ART).^6^ Individuals aged 18-years or older were recruited from 15 AIDS clinics in Brazil and stratified into two groups: those switching to DTG from a stable regimen without prior therapeutic failure (n = 373) and those who transitioned to DTG following failure of a previous ART regimen (n = 66). The mean age was 49.86-years (SD = 11.63) in the group without prior therapeutic failure and 47.05-years (SD = 11.11) in the group with prior ART regimen failure, with no statistically significant difference between the groups. Prior therapeutic failure was defined based on documented virologic failure recorded in clinical charts.

Sociodemographic, clinical, and behavioral data were collected at baseline. HRQoL was assessed using the RAND 36-Item Health Survey (RAND-36), which was administered at baseline and after 12-months of DTG-based treatment.^2^ The RAND 36-Item Health Survey (RAND-36) is a standardized instrument composed of 36-items that assess eight domains of health-related quality of life: physical functioning (10-items), role limitations due to physical health (4-items), role limitations due to emotional problems (three items), energy/fatigue (4-items), emotional well-being (5-items), social functioning (two items), bodily pain (2-items), and general health (5-items). In addition, a single item evaluates the individual’s self-perceived change in health over time. Only participants with complete HRQoL data at both time points were included in the longitudinal analysis.

Descriptive statistics were used to characterize the sample. Continuous variables were presented as means and standard deviations, whereas categorical variables were expressed as absolute frequencies and percentages. Differences in baseline characteristics across groups were evaluated using analysis of variance (ANOVA), followed by Bonferroni post hoc test.

To assess changes in RAND-36 domain scores from baseline to 12-months within each group, paired-sample *t*-tests were performed. Between-group comparisons of HRQoL changes were conducted using change scores (Δ), calculated as the difference between baseline and 12-month values, and compared using independent-samples *t*-tests. Effect sizes were estimated using Cohen’s *d* and interpreted as trivial (< 0.20), small (0.20–0.49), moderate (0.50–0.79), or large (≥ 0.80). A p-value < 0.05 was considered statistically significant.^7^

## Results

A total of 577 individuals were enrolled at baseline. Of these, 138 (23.9%) did not complete the 12-month follow-up and were excluded from the longitudinal analysis because of incomplete HRQoL data. The final analytical sample comprised 439 participants, of whom 373 (85.0%) switched to Dolutegravir (DTG)-based antiretroviral therapy from a stable ART regimen, without prior therapeutic failure (Group 1), and 66 (15.0%) switched to DTG following failure from a previous antiretroviral regimen (Group 2). Baseline sociodemographic characteristics and HIV risk factors are summarized in Table 1.

**Table 1:** Sample characterization and comparison between groups with and without prior therapeutic failure.

Variable	No Prior Failure (n = 373)	Prior Failure (n = 66)
Sex		
Male	257 (68.9%)	40 (60.6%)
Female	116 (31.1%)	26 (39.4%)
Education		
< 8-years	126 (33.8%)	36 (54.5%)
≥ 8-years	247 (66.2%)	30 (45.5%)
Race		
White	55 (14.7%)	8 (12.1%)
Black	118 (31.6%)	24 (36.4%)
Other	200 (53.6%)	34 (51.5%)
Income		
Other income/occupation	264 (70.8%)	46 (69.7%)
Employed	46 (12.3%)	10 (15.2%)
Retired	63 (16.9%)	10 (15.2%)
Risk Factors		
Male-to-male HIV risk: No	227 (60.9%)	52 (78.8%)
Male-to-male HIV risk: Yes	146 (39.1%)	14 (21.2%)
Bisexual HIV risk: No	308 (82.6%)	63 (95.5%)
Bisexual HIV risk: Yes	65 (17.4%)	3 (4.5%)
Heterosexual HIV risk: No	168 (45.0%)	18 (27.3%)
Heterosexual HIV risk: Yes	205 (55.0%)	48 (72.7%)
Injecting Drug Use: No	371 (99.5%)	66 (100.0%)
Injecting Drug Use: Yes	2 (0.5%)	0 (0.0%)
Hemotransfusion: No	369 (98.9%)	66 (100.0%)
Hemotransfusion: Yes	4 (1.1%)	0 (0.0%)
Hemophilia: No	372 (99.7%)	66 (100.0%)
Hemophilia: Yes	1 (0.3%)	0 (0.0%)
Occupational Exposure: No	369 (98.9%)	66 (100.0%)
Occupational Exposure: Yes	4 (1.1%)	0 (0.0%)

Post hoc Bonferroni comparisons showed that participants who did not complete the 12-month follow-up differed significantly from those receiving dolutegravir without prior therapeutic failure, presenting lower baseline HRQoL scores in role limitations due to physical health (p = 0.047), energy/fatigue (p = 0.003), emotional well-being (p = 0.005), pain (p = 0.016), and general health (p = 0.001), and were also younger (p = 0.001). In contrast, no consistent statistically significant differences were observed between participants lost to follow-up and those with prior therapeutic failure, suggesting a similar baseline profile between these groups.

Comparisons of RAND-36 domain scores at baseline and after 12-months are presented in Table 2, Table 3. Among participants without prior therapeutic failure (Group 1), no statistically significant changes were observed in any of the eight RAND-36 domains over the 12-month follow-up period. In contrast, among participants with prior therapeutic failure (Group 2), statistically significant improvements were observed in the Role-Emotional (Δ = 17.17; p = 0.010), Social Functioning (Δ = 10.79; p = 0.008), and Pain domains (Δ = 10.66; p = 0.024).

**Table 2:** Comparison of RAND-36 domains between groups with and without prior therapeutic failure.

	No prior therapeutic failure (n = 373)		Δ No Prior Failure	p-value	Prior therapeutic failure (n = 66)		Δ Prior Failure	p-value
	Mean ± SD Baseline	Mean ± SD Month 12			Mean ± SD Baseline	Mean ± SD Month 12		
Physical Functioning	82.24 ± 23.37	80.74 ± 24.53	-1.50	0.183	82.50 ± 22.18	82.05 ± 20.90	-0.45	0.848
Role-Physical	73.86 ± 36.07	70.71 ± 38.91	-3.15	0.120	63.26 ± 41.88	70.45 ± 38.71	7.19	0.182
Role-Emotional	74.08 ± 38.12	71.58 ± 39.75	-2.50	0.272	58.08 ± 43.49	75.25 ± 37.58	17.17	0.010
Energy/Fatigue	67.86 ± 21.70	66.66 ± 21.83	-1.20	0.276	63.64 ± 24.36	63.41 ± 23.26	-0.23	0.946
Emotional Well-Being	71.69 ± 23.15	70.14 ± 22.95	-1.55	0.212	66.18 ± 24.90	66.06 ± 25.81	-0.12	0.976
Social Functioning	77.11 ± 26.52	77.81 ± 23.01	0.69	0.629	70.08 ± 29.65	80.87 ± 24.01	10.79	0.008
Pain	76.38 ± 26.27	75.05 ± 25.50	-1.33	0.308	65.57 ± 28.42	76.23 ± 26.04	10.66	0.024
General Health	72.37 ± 18.75	71.33 ± 18.28	-1.04	0.261	60.68 ± 23.02	65.00 ± 19.45	4.32	0.084

**Table 3:** Between-group comparison of changes (Δ) in RAND-36 domain scores over 12-months in individuals with and without prior therapeutic failure.

RAND-36 Domain	Δ No Prior Failure (Mean ± SD)	Δ Prior Failure (Mean ± SD)	p-value	Cohen’s d	Interpretation
Physical Functioning	-1.50 ± 21.74	-0.45 ± 19.25	0.72	0.05	Trivial
Role-Physical	-3.15 ± 39.04	7.20 ± 43.36	0.06	0.27	Small-moderate
Role-Emotional	-2.50 ± 43.92	17.17 ± 52.38	<0.001	0.45	Moderate
Energy/Fatigue	-1.19 ± 21.11	-0.23 ± 27.39	0.79	0.04	Trivial
Emotional Well-Being	-1.54 ± 23.86	-1.12 ± 29.33	0.91	0.02	Trivial
Social Functioning	0.70 ± 28.13	10.80 ± 32.14	0.02	0.36	Moderate
Pain	-1.33 ± 25.26	8.60 ± 30.15	0.01	0.38	Moderate
General Health	-1.05 ± 17.95	4.32 ± 19.96	0.04	0.29	Small

**Fig. 1:**
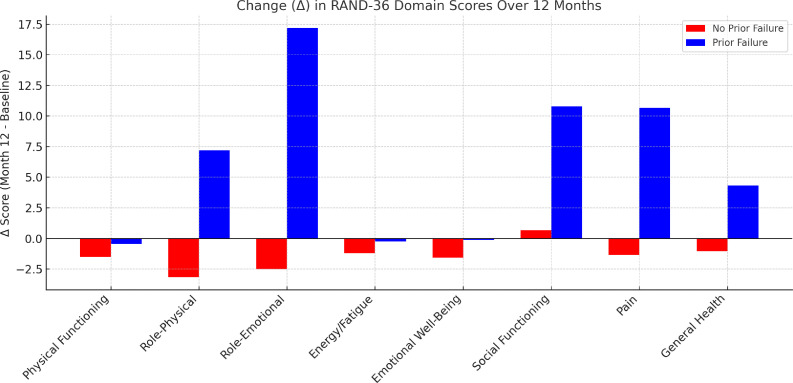
Change (Δ) in RAND-36 domain scores from baseline to 12-month follow-up among participants with and without prior therapeutic failure.

Mean changes (Δ) in RAND-36 domain scores from baseline to 12-months in both groups are illustrated in Fig. 1. Participants without prior therapeutic failure showed stable or slightly reduced scores across most domains, whereas participants with prior therapeutic failure demonstrated positive changes in the domains with statistically significant differences.

Between-group comparisons of change scores (Δ) demonstrated significantly greater improvements among individuals with prior therapeutic failure in the domains of role limitations due to emotional problems (p < 0.001), social functioning (p = 0.02), pain (p = 0.01), and general health (p = 0.04). A trend toward greater improvement was also observed in role-physical functioning (p = 0.06). No significant between-group differences were found for physical functioning (p = 0.72), energy/fatigue (p = 0.79), or emotional well-being (p = 0.91).

Effect sizes ranged from trivial to moderate, with the largest effect observed in the role-emotional domain (*d* = 0.45), followed by pain (*d* = 0.38) and social functioning (*d* = 0.36), indicating a consistent pattern of greater improvement in psychosocial domains among individuals with prior therapeutic failure.

## Discussion

This study assessed the impact of dolutegravir-based antiretroviral therapy (ART) on Health-Related Quality of Life (HRQoL) among people living with HIV, comparing individuals with and without prior therapeutic failure. While participants without a history of ART failure (switching from a stable regimen) exhibited stable HRQoL over the 12-month follow-up period, those who switched to dolutegravir following treatment failure demonstrated significant improvements in several domains, most notably role-emotional functioning, social functioning, and pain.

Our findings provide important insight into the pattern of loss to follow-up. Participants who did not complete the 12-month assessment showed a baseline profile comparable to those with prior therapeutic failure, while differing significantly from individuals who remained in care without prior failure. This suggests that attrition was not random, but rather associated with underlying clinical and functional vulnerability.

Gudala et al. reported HRQoL findings among people receiving ART in a South African tertiary-care setting, reinforcing the relevance of patient-reported outcomes when evaluating contemporary regimens.^8^ These findings are consistent with the improvements observed in the RAND-36 pain domain in the present study, suggesting that dolutegravir may enhance not only virological outcomes but also physical comfort and overall treatment tolerability. Similarly, data from a Swedish national registry showed a significant decline in self-reported side effects following the replacement of efavirenz with dolutegravir as the preferred third agent.^9^ That study further demonstrated that individuals reporting better physical and psychological health were significantly less likely to experience adverse effects, reinforcing the association between overall well-being and ART tolerability.

A four-year longitudinal analysis conducted in Cameroon reported significant improvements in mental and emotional health among patients initiating dolutegravir-based therapy.^5^ Likewise, a real-world study from India observed physical health gains among individuals who switched to dolutegravir.^4^ Together, these findings underscore the potential of dolutegravir to improve patient-reported outcomes across diverse clinical and sociocultural settings.

A cross-sectional study conducted in Ukraine showed lower treatment satisfaction among individuals receiving lopinavir-based regimens compared with those on dolutegravir-based therapy, even after adjustment for sociodemographic and clinical factors. The study also highlighted the influence of social determinants of health, including employment status, income, and substance use, on patient-reported outcomes.^10^ These observations support the notion that dolutegravir may contribute positively to the care experience of individuals with complex therapeutic histories. Brown et al. reported improvements in specific neuropsychiatric and functional symptoms, including feelings of sadness or depression, anxiety, concentration, sleep and dreams, energy levels, associated social impact, and the likelihood of being symptom free following initiation or switching to dolutegravir-containing regimens. The authors recommended favoring dolutegravir-containing first-line antiretroviral therapy.^11^ These findings strengthen the growing body of evidence suggesting that dolutegravir may positively influence psychological well-being, particularly in populations with prolonged or complicated treatment histories.

In the present study, individuals with prior therapeutic failure exhibited significantly greater improvements in role-emotional functioning, social functioning, and pain domains over the 12-month follow-up period. These findings are consistent with those of a recent Ugandan study, which identified poor adherence, largely driven by stigma, alcohol use, and forgetfulness, as the main determinant of virological failure among patients receiving dolutegravir-based regimens.^12^ Similarly, a Brazilian study reported better adherence among individuals using once-daily dolutegravir-based regimens, particularly when adequate counseling was provided, and there was no history of illicit drug use.^13^ In addition, greater improvements over time in emotional role functioning observed in the present analysis highlight the relevance of emotional well-being in treatment trajectories.

Some limitations should be considered when interpreting these findings. The number of participants with prior ART failure was relatively small, and information on the specific components of previous ART regimens was not available. Nevertheless, given that most people living with HIV in Brazil were receiving efavirenz-based therapy before failure, it is likely that the majority of participants in the failure group had been exposed to efavirenz. Despite these limitations, the sample size was sufficient to detect significant differences between groups.

## Conclusions

Dolutegravir-based therapy was associated with stable Health-Related Quality of Life (HRQoL) among individuals without prior antiretroviral therapy failure and with significant improvements in emotional, social, pain, and general health domains among those with a history of ART failure. These findings highlight the importance of incorporating patient-reported outcomes into the evaluation of treatment response and support the implementation of patient-centered care models that address both clinical outcomes and quality-of-life dimensions in people living with HIV.

## Institutional review board statement

The study was conducted in accordance with the Declaration of Helsinki and approved by the Research Ethics Committee of the Federal University of Bahia, Brazil (protocol code n°3.574.111, approval date: September 13^th,^ 2019).

## Informed consent statement

Informed consent was obtained from all subjects involved in the study.

## Data availability statement

The data supporting the findings of this study are available from the corresponding author upon reasonable request.

## Authors’ contributions

Conceptualization, L.L.-K. and C.B.; Methodology, L.L.-K., E.L., M.B.A., and C.B.; Formal analysis, L.L.-K.; Investigation, L.L.-K., E.L., M.B.A., F.B., M.V.G.L., A.R., and S.W.; Writing-original draft preparation, L.L.-K.; Writing-review and editing, L.L.-K., C.B., and K.P.; Supervision, C.B.; Project administration, L.L.-K. and C.B.; Funding acquisition, L.L.-K. All authors have read and agreed to the published version of the manuscript.

## Funding

This research was partially funded grant (#212964) from ViiV/GSK Brazil.

## Conflicts of interest

Carlos Brites has received a grant (#212964) from ViiV/GSK Brazil. For the remaining authors, none were declared.
